# Prevalence of HIV-associated nephropathy in children: a systematic review with meta-analysis of studies published between 2004 and 2019

**DOI:** 10.1007/s00467-026-07167-z

**Published:** 2026-02-06

**Authors:** Diogo Costa Garção, João Gabriel Santana Trindade, Susan Soares de Carvalho

**Affiliations:** 1 Universidade Federal de Sergipe, Av. Marechal Rondom, s/n, Jardim Rosa Elze, 49.100-000 São Cristóvão, SE Brazil; 2 Universidade Tiradentes, Aracaju, Brazil

**Keywords:** HIV-associated nephropathy, Antiretroviral therapy, Children, HIV, AIDS

## Abstract

**Introduction:**

Despite major advances in antiretroviral therapy, HIV infection remains a significant global public health challenge. HIV can directly affect the kidneys, leading to HIV-associated nephropathy in children, a condition characterized by proteinuria, elevated serum creatinine, and kidney enlargement. HIV-associated nephropathy is a serious complication that may progress to kidney failure and death.

**Objective:**

To estimate the prevalence of HIV-associated nephropathy in children.

**Methods:**

We searched PubMed, Embase, LILACS, SciELO, and Web of Science for relevant studies. Searches used the terms “AIDS-associated nephropathy” AND “child.”

**Results:**

A total of 1,181 records were identified, of which 10 studies (n = 1,136 children living with HIV) met inclusion criteria. Most included studies were conducted before widespread ART availability and used proteinuria as a proxy for HIV-associated nephropathy. The pooled prevalence of HIV-associated nephropathy was 17% (95% CI, 8%–31%; I^2^ = 93%, *p* < 0.01). Subgroup analysis showed marked geographic variation, with a prevalence of 29% (95% CI, 22%–38%) in Africa and 8% (95% CI, 3%–20%) in North America. By sex, 59% (95% CI, 49%–69%) of male children developed HIV-associated nephropathy compared with 41% (95% CI, 31%–51%) of female children. Boys were significantly more likely to develop HIV-associated nephropathy (*p* = 0.02). The mortality rate among affected children was 53% (95% CI, 40%–56%). Key risk factors included lack of antiretroviral therapy and the presence of AIDS.

**Conclusion:**

HIV-associated nephropathy was historically a common and life-threatening complication among children living with HIV. However, the available evidence is largely based on studies conducted more than a decade ago and often relied on proteinuria rather than biopsy-confirmed diagnosis.

## Introduction

HIV is a retrovirus that targets CD4 + T cells by hijacking their cellular machinery for replication, ultimately destroying these cells and progressively weakening the body’s ability to fight infections and disease [1, 2]. As CD4 cell counts decline, the immune system becomes increasingly compromised, leaving the body vulnerable to opportunistic infections and resulting in the development of AIDS [3].

Globally, approximately 40 million people are living with HIV, and an estimated 630,000 die each year from HIV-related complications [4]. Children account for about 6.5% of the worldwide HIV-positive population and are particularly susceptible to the systemic complications of the disease [5].

One of the most severe manifestations is HIV-associated nephropathy (HIVAN), a condition that can rapidly progress to chronic kidney disease, kidney failure, and death [6]. Although antiretroviral therapy (ART) has markedly reduced the burden of HIVAN, most prevalence data in children come from studies conducted before 2010. As a result, the applicability of these findings to children receiving contemporary ART regimens is limited.

Because of the substantial impact of HIVAN on pediatric health and the scarcity of recent global prevalence data, this review aims to estimate the prevalence of HIVAN in children and to identify potential risk factors associated with its development and progression.

## Methods

### Registration and guidelines

This review was conducted in accordance with the Preferred Reporting Items for Systematic Reviews and Meta-Analyses statement and the Cochrane Collaboration Handbook, version 6.1. A protocol was developed and registered in the PROSPERO database (CRD42024614080).

The PCO framework was used to formulate the research question, sharpen the focus of the review, and optimize evidence retrieval from the databases. The guiding question was: “What is the prevalence of HIVAN in children?” According to the PCO criteria: P (population) – children with HIVAN; C (comparison) – none; O (outcome) – prevalence.

### Search strategy

Five databases were searched to identify eligible studies: PubMed, Embase, LILACS, SciELO, and Web of Science. Searches were performed in October 2024 using the following English terms: “AIDS-Associated Nephropathy” AND “Child.”

### Study selection

Study selection was carried out in two stages. First, titles of all retrieved records were screened. Second, abstracts and full-text articles were assessed for eligibility. Studies were included if they reported the prevalence of HIVAN in children, irrespective of the diagnostic method used. Studies outside the scope of this review, case reports, case series, reviews, and letters to the editor were excluded.

### Data extraction

Data were extracted by one review author using standardized forms and independently checked by a second author. Extracted variables included the first author’s surname, geographic location of the study population, sample size, and the number of children diagnosed with HIVAN. Discrepancies were resolved through consultation with a third review author.

Diagnostic criteria for HIVAN varied widely across studies, reflecting the lack of standardized definitions in children. Only a minority of studies used kidney biopsy to confirm HIVAN; most relied on indirect markers such as proteinuria. These markers were frequently used as proxies for HIVAN because kidney biopsy is often not feasible in pediatric populations. For this review, we accepted the case definitions used by the original authors.

### Assessment of quality

Methodological quality was assessed using the revised JBI critical appraisal tool for prevalence studies [7], which comprises 9 items rated as “yes” or “no” to indicate potential risk of bias.

### Statistical analysis

Meta-analyses were performed using the “meta” package in R software [8]. Between-study heterogeneity was evaluated using Higgins’ I^2^ statistic. A 95% CI was applied, and statistical significance was set at p < 0.05. In addition to estimating the overall prevalence of HIVAN in children, subgroup analyses were conducted by geographic region and sex.

## Results

### Study selection process

**Fig. 1:**
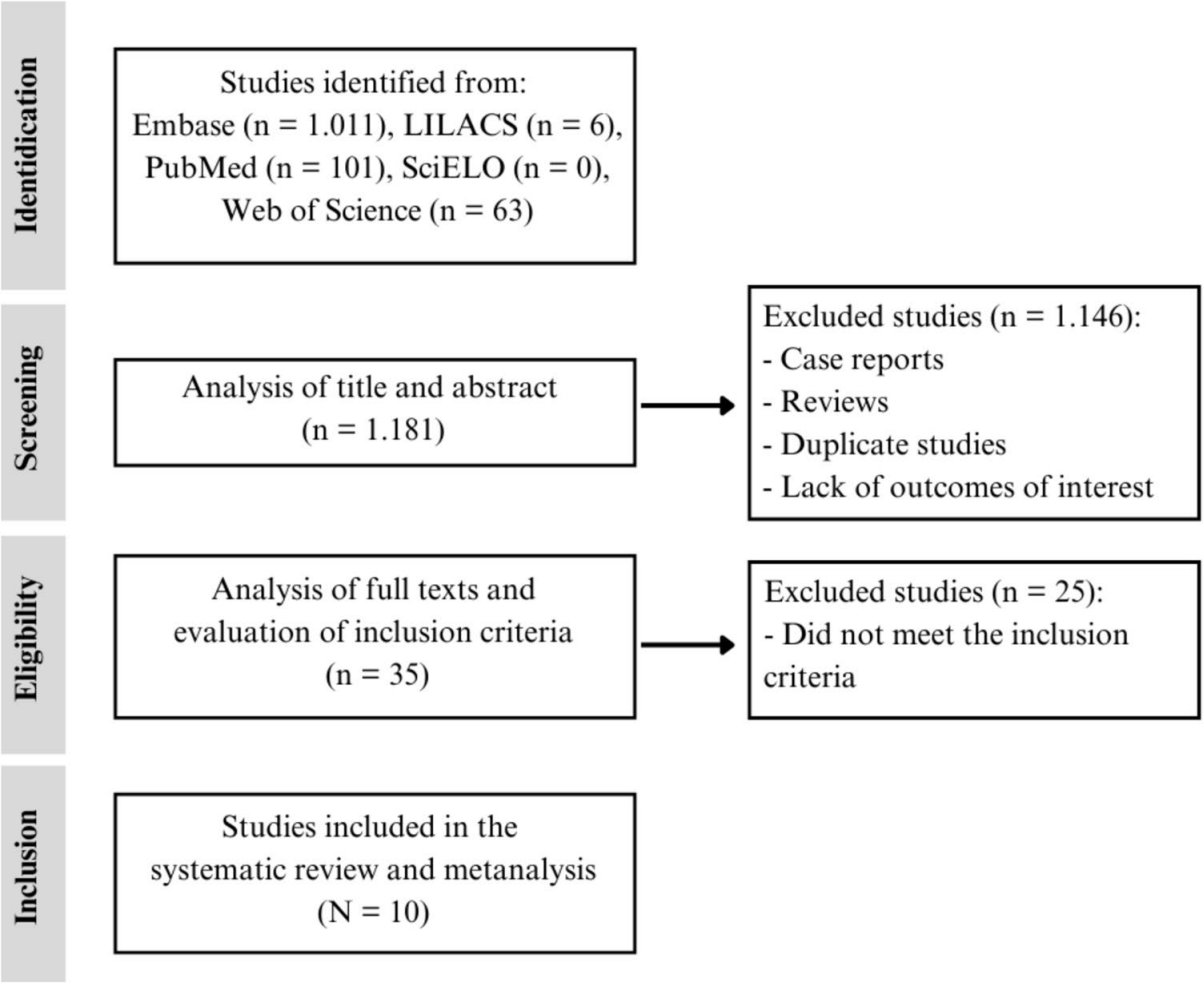
Flow diagram of the study selection process

A total of 1,181 studies were retrieved through database searches. Of these, 1,146 were excluded during the initial screening because they were case reports, reviews, duplicates, or did not report the outcome of interest. After full-text assessment, 25 additional studies were excluded for not meeting eligibility criteria. Ultimately, 10 studies were included in this review (Fig. 1).

### Assessment of risk of bias

Most included studies demonstrated a low risk of bias across all domains of the JBI checklist. However, three studies [9–11] were judged to have a high risk of bias for Domain 3, which evaluates whether the sample size was adequate to produce a reliable prevalence estimate (Table 1).

**Table 1:** Methodological quality assessment of the included studies using the JBI critical appraisal checklist for prevalence studies

Study ID	Tool domains								
	1st	2nd	3rd	4th	5th	6th	7th	8th	9th
Ahuja TS 2004	+	+	+	+	+	+	+	+	+
Anochie IC 2008	+	+	–	+	+	+	+	+	+
Chaparro AI 2008	+	+	+	+	+	+	+	+	+
Steel-Duncan J 2008	+	+	+	+	+	+	+	+	+
Miller ME 2009	+	+	–	+	+	+	+	+	+
Esezobor CI 2010	+	+	+	+	+	+	+	+	+
Ikpeme EE 2012	+	+	+	+	+	+	+	+	+
Coulibaly G 2013	+	+	+	+	+	+	+	+	+
Senguttuvan P 2014	+	+	–	+	+	+	+	+	+
Ibrahim HU 2019	+	+	+	+	+	+	+	+	+

### Study characteristics

A total of 10 studies were included in this review [9–18]. All were cross-sectional designs (four retrospective and six prospective) and together included 1,136 children living with HIV (Table 2). Although the search strategy did not restrict publication year, all eligible studies were published between 2004 and 2019.

**Table 2:** Characteristics of the included studies

Study ID	Study design	n	Continent
Ahuja TS 2004	Retrospective	60	North America
Anochie IC 2008	Prospective	10	Africa
Chaparro AI 2008	Retrospective	286	North America
Steel-Duncan J 2008	Prospective	196	North America
Miller ME 2009	Retrospective	18	North America
Esezobor CI 2010	Prospective	88	Africa
Ikpeme EE 2012	Prospective	98	Africa
Coulibaly G 2013	Prospective	122	Africa
Senguttuvan P 2014	Retrospective	8	Asia
Ibrahim HU 2019	Prospective	250	Africa

### Prevalence of HIVAN in children

In most included studies, HIVAN was identified based on persistent proteinuria and/or ultrasound abnormalities rather than histological confirmation. This limitation should be considered when interpreting the pooled prevalence estimates.

**Fig. 2:**
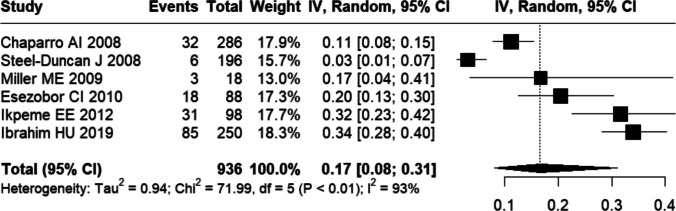
Forest plot of the prevalence of HIVAN in children

Of the 10 included studies, 6 reported data on the prevalence of HIVAN in children. Among 936 children living with HIV, 175 were diagnosed with HIVAN, yielding a pooled prevalence of 17% (95% CI, 8%–31%; I^2^ = 93%, *p* < 0.01), indicating a high prevalence of HIVAN in this population (Fig. 2).

Subgroup analyses revealed substantial geographic variation: the prevalence of HIVAN in Africa was significantly higher than in North America (*p* < 0.01). The pooled prevalence was 29% (95% CI, 22%–38%) in Africa and 8% (95% CI, 3%–20%) in North America.

Sex-stratified analysis showed that 59% (95% CI, 49%–69%) of boys developed HIVAN, compared with 41% (95% CI, 31%–51%) of girls. Boys were significantly more likely to develop HIVAN (OR = 2.89; 95% CI, 1.14–7.29; *p* = 0.02).

The pooled mortality rate among children with HIVAN was 53% (95% CI, 40%–56%), underscoring the lethality of this condition. Lack of ART was a prominent risk factor, observed in 66% (95% CI, 48%–81%) of affected children. Children not receiving ART were substantially more likely to develop HIVAN (OR = 4.54; 95% CI, 1.17–17.60; *p* = 0.03), consistent with the advanced disease severity associated with progression to AIDS.

## Discussion

This review examined the prevalence of HIVAN in children and found a high overall prevalence, particularly in Africa. The findings also indicate that boys are more likely than girls to develop HIVAN and that the condition is highly lethal, with mortality further increased in the absence of ART.

Previous studies have shown that nephropathy is a common manifestation of HIV infection in children. In a cross-sectional study of children with HIV aged 10 months to 17 years, albuminuria, microalbuminuria, and serum creatinine levels were measured, followed by calculation of glomerular filtration rate. All participants were receiving ART, and the prevalence of HIVAN in this cohort was 16.2% [19]. This result is consistent with the findings of the present review and supports the notion that HIVAN remains a frequent kidney complication in children with HIV, even among those on ART.

Other studies have also documented a high prevalence of HIVAN in pediatric populations. In a prospective study, serum creatinine, microalbuminuria, and kidney ultrasound findings were evaluated in children with HIV. HIVAN was diagnosed when persistent proteinuria was accompanied by abnormal kidney ultrasound findings, yielding a prevalence of 31% in this population [17].

Several mechanisms have been implicated in the development of kidney disease in children with HIV. Direct interaction of HIV with kidney cells, mediated by viral proteins, stimulates the release of inflammatory cytokines and leads to a spectrum of pathological kidney lesions. These processes, together with activation of the RAAS, contribute to proteinuria and focal segmental glomerulosclerosis, which are characteristic of HIVAN. Collectively, these mechanisms are thought to underlie the high prevalence of nephropathy observed in children living with HIV [5, 6, 20]. However, the contribution of the RAAS remains speculative and is supported by limited evidence.

Studies consistently report a high prevalence of nephropathy in children with HIV, although rates vary worldwide [14]. One possible explanation for this variation is the requirement for kidney biopsy to confirm HIVAN [21]. Because biopsy is invasive and costly, most studies instead rely on proteinuria and albuminuria as noninvasive screening markers to identify children with suspected nephropathy.

In a multicenter study from South Africa, kidney biopsies were performed in children with HIV who had been clinically diagnosed with nephropathy based on urinary albumin or protein-to-creatinine ratio. Histological examination confirmed HIVAN in 26.9% of these clinically suspected cases [22]. This finding illustrates that kidney biopsy improves diagnostic accuracy by confirming histopathological changes consistent with the clinical presentation. Nevertheless, the invasive nature and complexity of biopsy, particularly in children, limit its global use for diagnosing HIVAN and may contribute to overestimation of prevalence when diagnosis is based solely on laboratory markers.

More detailed analyses suggest that some ethnic groups and populations living in regions with a high HIV burden may be more vulnerable to severe complications, including HIVAN [23]. In this context, comparisons across continents show a higher prevalence of HIVAN among children in Africa, where studies of HIV-related kidney disease have reported prevalence rates above 30% [16, 17]. In contrast, prevalence as low as 3% has been reported among children in North America [18]. These findings are in line with the results of the present review and underscore substantial geographic disparities in the prevalence of HIVAN in children.

One possible explanation for the higher prevalence of HIVAN in Africa is the presence of genetic factors associated with African ancestry [24]. Specifically, this genetic predisposition has been linked to the *APOL1* gene and its two risk alleles, G1 and G2, as the virus is thought to exploit this protein to induce kidney disease and trigger the development of HIVAN [6, 25, 26]. However, the precise mechanism by which *APOL1* contributes to abnormal kidney function in individuals with HIV remains poorly understood. It is hypothesized that HIV-related inflammation stimulates cytokine release, which drives overexpression of *APOL1* and its risk variants [27]. These alleles are believed to exert direct cytotoxic effects on podocytes, disrupting the integrity of the glomerular filtration barrier and resulting in proteinuria [28].

This association was first demonstrated by Kopp et al., who determined *APOL1* genotypes in patients diagnosed with either primary focal segmental glomerulosclerosis or HIVAN as part of a multicenter genetic study conducted in the United States. In addition, the study examined genotypes from individuals belonging to different ethnic groups classified by the Human Genome Diversity Project [29]. Under a recessive model, the *APOL1* risk variants were shown to confer nearly a 30-fold increase in the odds of developing HIVAN. Global population analyses confirmed that these risk alleles are found exclusively on chromosomes of individuals with African ancestry.

Further evidence indicates that children of African descent are more susceptible to developing HIVAN. A retrospective study from a North American hospital evaluated children and adolescents with HIV and found that most participants were of African descent. Nearly half exhibited intermittent or persistent proteinuria, and all cases of HIVAN occurred exclusively in these individuals [30].

In addition to genetic susceptibility, socioeconomic disparities between regions such as North America and Africa may contribute to the higher prevalence of HIVAN in children of African descent. Sub-Saharan Africa faces substantial socioeconomic challenges, including limited access to health care services and high rates of childhood malnutrition. These factors further impair the immune system, increasing vulnerability to opportunistic infections and HIV-related complications such as HIVAN [28].

Consistent with the present review, Ikobo et al. reported a predominance of HIVAN among male children living with HIV in the Republic of the Congo, located in sub-Saharan Africa [31]. However, this sex-based difference has not been observed in other studies, which generally suggest that boys and girls are equally susceptible to HIVAN [16]. The reasons for these discrepancies remain unclear but may reflect inter-regional and intercultural differences in health-care-seeking behaviors [12]. For instance, in parts of Nigeria, cultural and religious norms may limit health-care access for girls, potentially contributing to underdiagnosis among female patients [32].

The typical clinical presentation of HIVAN is characterized by massive proteinuria within the context of nephrotic syndrome, which can progress to kidney failure in advanced stages of HIV infection and ultimately lead to death [33]. Findings from the present review suggest that ART can mitigate the onset and progression of HIVAN. Previous studies have shown that individuals living with HIV who are not receiving ART are at substantially greater risk of developing HIVAN [29]. In a retrospective study of nephropathy in HIV-infected children undergoing ART, most children with elevated proteinuria levels showed improvement following ART initiation [13]. ART improves prognosis by suppressing viral replication, limiting kidney injury induced by viral proteins, and restoring immune function, as evidenced by rising CD4 + T-cell counts.

In a retrospective study, Lescure et al. analyzed clinical and histopathological data from HIV-infected patients with biopsy-confirmed glomerular disease and found a strong association between low CD4 + T-cell counts, high viral load, and the presence of HIVAN [34]. Similarly, progression to AIDS has been identified as a major risk factor for HIVAN in children [5]. This may be explained by the fact that increased viral replication, combined with progressive immune system deterioration, heightens the likelihood of viral proteins inducing kidney injury. This contributes to the development of severe nephropathy with rapid progression to kidney failure, a condition associated with poor survival outcomes [18, 20].

### Limitations of study

This review has several limitations that should be acknowledged. First, most included studies were conducted more than a decade ago, before the widespread use of contemporary ART regimens. Consequently, the findings largely reflect historical data and may not accurately represent the current prevalence of HIVAN in children receiving modern therapy. Second, diagnostic criteria for HIVAN were heterogeneous. Most studies relied on persistent proteinuria and other indirect markers rather than biopsy-confirmed diagnoses. Because proteinuria is nonspecific and may reflect other HIV-related kidney diseases, such as immune complex glomerulonephritis, this approach may have led to overestimation of the true prevalence of HIVAN. Third, the small number of eligible studies, drawn from only a few geographic regions, contributed to the substantial heterogeneity observed and limited broader comparisons across continents. Finally, none of the included studies incorporated genetic data, particularly *APOL1* risk variants, which are major determinants of kidney disease susceptibility in populations of African ancestry. The absence of these data restricts the ability to account for genetic predisposition in the prevalence estimates reported here.

## Conclusion

This systematic review and meta-analysis provides a quantitative synthesis of the available evidence on the prevalence of HIVAN in children. The pooled data suggest that HIVAN was historically a frequent and severe complication, particularly among children in Africa, boys, and those not receiving ART. However, most included studies were conducted more than a decade ago and relied on proteinuria and other nonspecific markers rather than biopsy-confirmed diagnoses, which may have overestimated the true prevalence of HIVAN. In addition, none of the studies incorporated genetic information such as *APOL1* risk variants, which are key determinants of susceptibility.

Taken together, these limitations indicate that the findings should be interpreted primarily as reflective of historical data, with limited applicability to the current ART era. Despite these constraints, this review underscores the clinical relevance of HIVAN in children and highlights the urgent need for contemporary, well-designed studies that use standardized diagnostic criteria and include genetic risk assessment to more accurately define the burden of HIVAN in children worldwide.

## Data Availability

Data supporting the findings of this study are available from the corresponding author, Diogo Costa Garção, upon reasonable request.

## References

[1] Cachay ER (2024) Human immunodeficiency virus (HIV) infection. MSD Manual. https://www.msdmanuals.com/home/infections/human-immunodeficiency-virus-hiv-infection/human-immunodeficiency-virus-hiv-infection

[2] Fry SHL, Barnabas SL, Cotton MF (2019) Tuberculosis and HIV—An update on the “cursed duet” in children. Front Pediatr 7:159 32211351 10.3389/fped.2019.00159 PMC7073470

[3] Trickey A, McGinnis K, Gill MJ, Abgrall S, Berenguer J, Wyen C, Hessamfar M, Reiss P, Kusejko K, Silverberg MJ, Imaz A, Teira R, d’Arminio Monforte A, Zangerle R, Guest JL, Papastamopoulos V, Crane H, Sterling TR, Grabar S, Ingle SM, Sterne JAC (2024) Longitudinal trends in causes of death among adults with HIV on antiretroviral therapy in Europe and North America from 1996 to 2020: a collaboration of cohort studies. Lancet HIV 11:e176–e185. 10.1016/S2352-3018(23)00272-2 38280393 10.1016/S2352-3018(23)00272-2 PMC11656032

[4] UNAIDS (2024) UNAIDS global AIDS update 2024. https://www.unaids.org/en/resources/documents/2024/global-aids-update-2024. Accessed 17 Aug 2025

[5] Nkoy AB, Ekulu PM, Labarque V, Van Den Heuvel LP, Levtchenko EN (2023) HIV-associated nephropathy in children: challenges in a resource-limited setting. Pediatr Nephrol 38:2509–2521. 10.1007/s00467-022-05819-4 36472655 10.1007/s00467-022-05819-4

[6] Ray PE, Li J, Das J, Xu L, Yu J, Han Z (2024) Pathogenesis of HIV associated nephropathy in children and adolescents: taking a hard look 40 years later in the era of gene-environmental interaction. Am J Physiol-Renal Physiol 327:F1049–F1066. 10.1152/ajprenal.00208.2024 39323389 10.1152/ajprenal.00208.2024 PMC11687833

[7] Munn Z, Moola S, Lisy K, Riitano D, Tufanaru C (2015) Methodological guidance for systematic reviews of observational epidemiological studies reporting prevalence and cumulative incidence data. Int J Evid Based Healthc 13:147–153. 10.1097/XEB.0000000000000054 26317388 10.1097/XEB.0000000000000054

[8] Balduzzi S, Rücker G, Schwarzer G (2019) How to perform a meta-analysis with R: a practical tutorial. Evid Based Ment Health 22:153–160. 10.1136/ebmental-2019-300117 31563865 10.1136/ebmental-2019-300117 PMC10231495

[9] Anochie IC, Eke FU, Okpere AN (2008) Human immunodeficiency virus-associated nephropathy (HIVAN) in Nigerian children. Pediatr Nephrol 23:117–122. 10.1007/s00467-007-0621-0 17985161 10.1007/s00467-007-0621-0

[10] Miller MEY, Williams JA (2009) Chronic renal failure in Jamaican children—an update (2001–2006). West Indian Med J 58:231–234 20043530

[11] Senguttuvan P, Gowtham S, Soundararajan P (2014) Human immunodeficiency virus-associated nephropathy (HIVAN) in Indian children. Open Urol Nephrol J 7:105–107. 10.2174/1874303X01407010105

[12] Ahuja TS, Abbott KC, Pack L, Kuo YF (2004) HIV-associated nephropathy and end-stage renal disease in children in the United States. Pediatr Nephrol 19:808–811. 10.1007/s00467-004-1482-4 15141343 10.1007/s00467-004-1482-4

[13] Chaparro AI, Mitchell CD, Abitbol CL, Wilkinson JD, Baldarrago G, Lopez E, Zilleruelo G (2008) Proteinuria in children infected with the human immunodeficiency virus. J Pediatr 152:844–849 18492529 10.1016/j.jpeds.2007.11.007

[14] Coulibaly G, Kouéta F, Ouédraogo O, Dao L, Lengani A, Yé D (2013) Prevalence of proteinuria in children followed-up for HIV infection at Pediatric University Hospital Charles-de-Gaulle (CHUP-CDG) of Ouagadougou. Bull Soc Pathol Exot 106:13–17. 10.1007/s13149-012-0270-9 23315307 10.1007/s13149-012-0270-9

[15] Esezobor CI, Iroha E, Onifade E, Akinsulie AO, Temiye EO, Ezeaka C (2010) Prevalence of proteinuria among HIV-infected children attending a tertiary hospital in Lagos, Nigeria. J Trop Pediatr 56:187–190. 10.1093/tropej/fmp090 19793893 10.1093/tropej/fmp090

[16] Ibrahim H, Elechi H, Rabasa A, Ashir G, Farouk A, Yauba M, Ibrahim B (2019) Prevalence and pattern of human immunodeficiency virus-associated nephropathy among human immunodeficiency virus-positive children at the University of Maiduguri Teaching Hospital, Nigeria. Saudi J Kidney Dis Transpl 30:843. 10.4103/1319-2442.265460 31464241 10.4103/1319-2442.265460

[17] Ikpeme EE, Ekrikpo UE, Akpan MU, Ekaidem SI (2012) Determining the prevalence of human immunodeficiency virus-associated nephropathy (HIVAN) using proteinuria and ultrasound findings in a Nigerian paediatric HIV population. Pan Afr Med J 11:13 22368756 PMC3283028

[18] Steel-Duncan J, Miller M, Pierre RB, Dunkley-Thompson J, Palmer P, Evans-Gilbert T, Rodriquez B, Christie CDC, Kingston Paediatric and Perinatal HIV/AIDS Study Group (2008) Renal manifestations in HIV-infected Jamaican children. West Indian Med J 57:246–252 19583123

[19] Iduoriyekemwen N, Sadoh W, Sadoh A (2013) Prevalence of renal disease in Nigerian children infected with the human immunodeficiency virus and on highly active antiretroviral therapy. Saudi J Kidney Dis Transpl 24:172. 10.4103/1319-2442.106364 23354220 10.4103/1319-2442.106364

[20] Ray PE, Li J, Das JR, Tang P (2021) Childhood HIV-associated nephropathy: 36 years later. Pediatr Nephrol 36:2189–2201. 10.1007/s00467-020-04756-4 33044676 10.1007/s00467-020-04756-4 PMC8061423

[21] Jindal AK, Tiewsoh K, Pilania RK (2018) A review of renal disease in children with HIV infection. Infect Dis 50:1–12 10.1080/23744235.2017.1371852 28885079

[22] Ramsuran D, Bhimma R, Ramdial PK, Naicker E, Adhikari M, Deonarain J, Sing Y, Naicker T (2012) The spectrum of HIV-related nephropathy in children. Pediatr Nephrol 27:821–827. 10.1007/s00467-011-2074-8 22205506 10.1007/s00467-011-2074-8

[23] Dondo V, Mujuru HA, Nathoo KJ, Chirehwa M, Mufandaedza Z (2013) Renal abnormalities among HIV-infected, antiretroviral naive children, Harare, Zimbabwe: a cross-sectional study. BMC Pediatr 13:75. 10.1186/1471-2431-13-75 23663553 10.1186/1471-2431-13-75 PMC3654941

[24] Ekulu PM, Nkoy AB, Adebayo OC, Kazadi OK, Aloni MN, Arcolino FO, Ngiyulu RM, Gini JL, Lepira FB, Van Den Heuvel LP, Levtchenko EN (2021) A focus on the association of Apol1 with kidney disease in children. Pediatr Nephrol 36:777–788. 10.1007/s00467-020-04553-z 32253519 10.1007/s00467-020-04553-z

[25] Bookholane H, Wearne N, Surapaneni A, Ash S, Berghammer-Böhmer R, Omar A, Spies R, Grams ME (2020) Predictors and prognosis of HIV-associated nephropathy on kidney biopsy in South Africa. Kidney Int Rep 5:1799–1804. 10.1016/j.ekir.2020.06.036 33102974 10.1016/j.ekir.2020.06.036 PMC7569688

[26] Zhu JY, Fu Y, van de Leemput J, Yu Y, Li J, Ray PE, Han Z (2024) HIV-1 Nef acts in synergy with APOL1-G1 to induce nephrocyte cell death in a new *Drosophila* model of HIV-related kidney diseases. bioRxiv. 10.1101/2024.03.08.58406939803578

[27] Goyal R, Singhal PC (2021) *APOL1* risk variants and the development of HIV-associated nephropathy. FEBS J 288:5586–5597. 10.1111/febs.15677 33340240 10.1111/febs.15677 PMC8213861

[28] Kasembeli AN, Duarte R, Ramsay M, Mosiane P, Dickens C, Dix-Peek T, Limou S, Sezgin E, Nelson GW, Fogo AB, Goetsch S, Kopp JB, Winkler CA, Naicker S (2015) *APOL1* risk variants are strongly associated with HIV-associated nephropathy in black South Africans. J Am Soc Nephrol 26:2882–2890. 10.1681/ASN.2014050469 25788523 10.1681/ASN.2014050469 PMC4625661

[29] Kopp JB, Nelson GW, Sampath K, Johnson RC, Genovese G, An P, Friedman D, Briggs W, Dart R, Korbet S, Mokrzycki MH, Kimmel PL, Limou S, Ahuja TS, Berns JS, Fryc J, Simon EE, Smith MC, Trachtman H et al (2011) *APOL1* genetic variants in focal segmental glomerulosclerosis and HIV-associated nephropathy. J Am Soc Nephrol 22:2129–2137. 10.1681/ASN.2011040388 21997394 10.1681/ASN.2011040388 PMC3231787

[30] Beng H, Rakhmanina N, Moudgil A, Tuchman S, Ahn SY, Griffith C, Mims MM, Ray PE (2020) HIV-associated CKDs in children and adolescents. Kidney Int Rep 5:2292–2300. 10.1016/j.ekir.2020.09.001 33305123 10.1016/j.ekir.2020.09.001 PMC7710839

[31] Ikobo LCO, Ibara RBO, Ngakengni NY, Babomi L, Bowassa GE, Ngamo LT, Mandilou SVM, Bouithy SN, Nika ER, Babela JRM (2020) Proteinuria in children living with HIV on highly active antiretroviral therapy (HAART). Open J Pediatr 10:255–262. 10.4236/ojped.2020.102026

[32] Agaba EI, Agaba PA, Sirisena ND, Anteyi EA, Idoko JA (2003) Renal disease in the acquired immunodeficiency syndrome in north central Nigeria. Niger J Med 12:120–125 14737980

[33] Rivera FB, Ansay MFM, Golbin JM, Alfonso PGI, Mangubat GFE, Menghrajani RHS, Placino S, Taliño MKV, De Luna DV, Cabrera N, Trinidad CN, Kazory A (2022) HIV-associated nephropathy in 2022. Glomerular Dis 3:1–11. 10.1159/000526868 36816427 10.1159/000526868 PMC9936764

[34] Lescure FX, Flateau C, Pacanowski J, Brocheriou I, Rondeau E, Girard PM, Ronco P, Pialoux G, Plaisier E (2012) HIV-associated kidney glomerular diseases: changes with time and HAART. Nephrol Dial Transplant 27:2349–2355. 10.1093/ndt/gfr676 22248510 10.1093/ndt/gfr676

